# 同胞全相合造血干细胞移植治疗极重型再生障碍性贫血合并抗磷脂综合征一例

**DOI:** 10.3760/cma.j.issn.0253-2727.2021.12.018

**Published:** 2021-12

**Authors:** 士源 周, 倩 朱, 静 李, 超 马, 思璐 刘, 斌 顾, 骁 马, 小津 吴, 德沛 吴

**Affiliations:** 1 苏州大学附属第一医院，苏州 215006 The First Affiliated Hospital of Soochow University, Suzhou 215006, China; 2 苏州弘慈血液病医院，苏州 215006 Soochow Hopes Hematology Hospital, Suzhou 215006, China

患者，女，29岁，因“发热伴皮肤瘀点2周，伴偏瘫1周”入院。患者于2周前无明显诱因出现鼻腔黏膜和齿龈出血，伴发热（最高体温39 °C）、头晕及乏力，当地医院化验示全血细胞减少，随后出现头痛伴左侧肢体偏瘫及右侧眼裂变小。当地医院予抗感染、血制品输注支持及降颅压等处理后热退，但血小板计数进行性下降，遂转诊我院。既往史：院外体检提示“桥本甲状腺炎”，无特殊用药史。平素月经正常，无复发性流产史。家族无易栓症史。入院体检：体温正常，血压、脉搏稳定，贫血貌，皮肤、黏膜散在瘀点、瘀斑，右侧眼裂明显偏小，右侧瞳孔偏小，伸舌左偏，左上肢肌力3级，右上肢肌力5级，双下肢肌力3级，双侧Babinski征（+）。辅助检查：血常规：WBC 0.78×10^9^/L，中性粒细胞0.15×10^9^/L，RBC 2.16×10^12^/L，网织红细胞2.6×10^9^/L，HGB 65 g/L，PLT 0×10^9^/L，淋巴细胞80％，未见原始幼稚细胞。淋巴细胞亚群：CD4/CD8＝0.7。骨髓象：有核细胞增生极度减低，未见明显病态造血。骨髓活检：骨髓造血岛中非造血细胞多见，印象：再生障碍性贫血。流式细胞术分析、白血病相关融合基因、染色体核型分析、二代基因测序均未见异常。头颅CT平扫：右侧半卵圆区低密度灶。磁共振（MRI）：右侧额叶、胼胝体及右侧半卵圆中心区异常信号影，考虑梗死伴梗死后少量渗血。磁共振血管造影（MRA）：双侧颈内动脉各段纤细，并见局限性狭窄，左侧大脑中动脉M段节段性闭塞，血栓可能大，双侧大脑后动脉P1段纤细。蛋白C、蛋白S均正常。阵发性睡眠性血红蛋白尿症（PNH）筛查（CD55/CD59和FLAER检查）未见异常。红细胞沉降率第1小时139 mm。自身抗体谱：抗核抗体1∶320（+）、均质型，抗SS-A抗体52KD（+）。抗心磷脂抗体（+），抗β2-GP-1（+）。患者及其胞弟HLA配型全相合。HLA抗体筛查：Ⅰ类强阳性［多个位点平均荧光强度（MFI值）>8000，其中A26位点MFI值为10 527，Cw5位点14 921，Cw8位点14 118］，Ⅱ类阳性。诊断：极重型再生障碍性贫血（VSAA）、脑梗死、抗磷脂综合征、桥本甲状腺炎。入院后予以足量甲泼尼龙、环孢素A治疗2周，并予静脉免疫球蛋白输注0.4 g·kg^−1^·d^−1^×5 d，同时予促造血、预防感染、血制品输注、脱水降颅压、改善颅内微循环等治疗。患者对无关供者来源血小板输注反应极差，仅对其胞弟和父亲来源血小板有反应。经治疗后，患者神经系统症状短暂改善，但入院后10 d再次出现头痛、呕吐、半身肢体肌力下降，头颅MRI提示新发蛛网膜下腔出血。给予足量免疫抑制及促造血治疗，患者血象无改善，中性粒细胞持续<0.1×10^9^/L，血小板计数持续<10×10^9^/L但患者神经系统症状再次好转。供者（胞弟）移植前体检，未发现明显异常，其中抗核抗体全套及抗磷脂抗体筛查均为阴性。经CD20单抗及血浆置换清扫HLA抗体后，患者进入移植预处理程序：氟达拉滨30 mg·m^−2^·d^−1^（−6 d～−2 d）；环磷酰胺50 mg·kg^−1^·d^−1^（−3、−2 d）；兔抗人胸腺细胞免疫球蛋白2.5 mg·kg^−1^·d^−1^（−6 d～−2 d），于2020年6月行同胞全相合造血干细胞移植，回输单个核细胞16.63×10^8^/kg，CD34^+^细胞5.49×10^6^/kg，CD3^+^细胞1.85×10^8^/kg。+10 d中性粒细胞植入，+13 d血小板植入。移植后发生癫痫发作，检测环孢素A血药浓度偏高，予调低环孢素A剂量后未再发作。移植当天干细胞回输前及+20 d复查HLA抗体，预处理前检出的多个位点HLA抗体MFI值下降，至+20 d时MFI值已降至8000以下（A26位点为2929，Cw5位点为7881，Cw8位点为5334）。未发生移植物抗宿主病（GVHD）、巨细胞病毒感染等并发症，MRI示头颅原病灶稳定吸收，于移植后1个月出院。出院后继续服用环孢素A并口服阿司匹林抗血小板治疗。随访至移植后6个月，血象稳定，供者嵌合度良好，抗核抗体、抗心磷脂抗体和抗β2-GP1仍低滴度阳性，未再发生神经系统症状。

